# Japanese reproductive medicine under health insurance: A brief review and prospects

**DOI:** 10.1002/rmb2.12646

**Published:** 2025-04-03

**Authors:** Akira Iwase

**Affiliations:** ^1^ Department of Obstetrics and Gynecology Gunma University Graduate School of Medicine Maebashi Japan

In 2020, the Japanese government introduced measures to reduce the financial burden of infertility treatment and create an environment that facilitates access to care, positioning these efforts as part of the government strategy to combat declining birthrates. Simultaneously, the government began to consider a shift from the conventional subsidy system to coverage through the national health insurance. The treatments included assisted reproductive technologies (ART), related medications, and artificial insemination. Before insurance coverage, under a specific fertility treatment support program, subsidies were provided based on the amount spent on procedures, with restrictions on eligible ages and the number of treatments. The insurance coverage review did not include reproductive treatments involving third parties or fertility preservation treatments for patients with cancer.

Before the introduction of insurance coverage, the Japanese Society for Reproductive Medicine, in cooperation with the Japanese Society of Obstetrics and Gynecology and the Japanese Urological Association, began preparing clinical guidelines. ART has a shorter history than other medical fields and is often introduced without sufficient evidence. However, the guidelines were created to gather as much evidence as possible in a short period and cover a wide range of topics, including indication criteria, ovarian stimulation methods, embryo culture and manipulation, embryo transfer, add‐on treatments, and male infertility.^1^ These guidelines provide evidence levels and recommendations for each treatment method, which are referred to as the insurance coverage criteria. A survey conducted before the introduction of insurance identified that the widely used add‐on treatments included assisted hatching (85.1%), chronic endometritis testing and treatment (77.2% and 76.9%, respectively), artificial oocyte activation (67.3%), and endometrial receptivity testing (64.2%).^2^ However, only some of these treatments are covered by insurance based on recommendations in the guidelines.

The Japan Society of Obstetrics and Gynecology annually registers and analyzes ART treatment cycles. In April 2020, following the coronavirus disease 2019 (COVID‐19) outbreak, the Japanese Society for Reproductive Medicine issued a statement recommending the postponement of ART and temporarily reducing the number of procedures performed.^3^ In 2021, the number of ART cases reached 498 140, an increase of 10.7% from the previous year, with the number of births being 69 797 (15.5% increase).^4^ This increase was presumably related to the expansion of subsidies prior to the introduction of insurance coverage and the resumption of treatments previously postponed owing to COVID‐19. Furthermore, in 2022, in the first year of insurance coverage, the number of treatment cycles and births reached 543 630 and 77 206, respectively, both of which were significantly higher than those in the previous year.^5^ Freeze–thaw embryo transfers were particularly common, with 264 412 procedures performed. Although the number of procedures performed increased in rural areas and at small facilities due to the reduced financial burden, a survey by the committee of the Japan Society of Obstetrics and Gynecology discovered that a relatively large number of major facilities continued to offer uninsured care. Moreover, the number of egg retrieval procedures did not change significantly after the introduction of insurance coverage.^6^

Guidelines play a crucial role for medical practitioners involved in reproductive medicine by helping them provide appropriate care based on the latest evidence. In this era of insurance coverage, these guidelines serve as a foundation for standard medical practices. Even though insurance coverage is expected to facilitate the accumulation of treatment data and further standardize practices, the flexibility of tailoring treatments to individual patient preferences may become limited. Therefore, guidelines must be revised based on the latest evidence. The revision of the reproductive medicine guidelines in Japan is currently in the final stages, and the updated version is scheduled for publication this year.

In Japan, a system of advanced medical care exists that refers to medical technologies under evaluation by the Minister of Health, Labor, and Welfare to determine whether they should be covered by the national health insurance. Several reproductive technologies, including add‐on treatments, that were initially given a relatively low recommendation level in the first edition of the guidelines, are currently being implemented in advanced medical care. Data on advanced medical care will be compiled at appropriate stages and reflected in the guidelines, either as newly listed technologies or as modifications to recommendations for previously listed technologies. These changes are linked to the inclusion of these technologies in insurance coverage.

## CONFLICT OF INTEREST STATEMENT

The author declares that the research was conducted in the absence of any commercial or financial relationships that could be construed as a potential conflict of interest. Akira Iwase is the Editor‐in‐Chief of Reproductive Medicine and Biology and a co‐author of this article. To minimize bias, he was excluded from all editorial decision‐making related to the acceptance of this article for publication.

## Data Availability

Data sharing not applicable to this article as no datasets were generated or analysed during the current study.
